# Antimicrobial Resistance Profile of Enterococcal Isolates From Clinical Specimens at a Tertiary Care Hospital in Western Maharashtra, India

**DOI:** 10.7759/cureus.73416

**Published:** 2024-11-10

**Authors:** Sourish Hota, Satish R Patil, Priyanka M Mane

**Affiliations:** 1 Department of Microbiology, Krishna Institute of Medical Sciences, Krishna Vishwa Vidyapeeth (Deemed to Be University), Karad, IND

**Keywords:** antimicrobial resistance, bact-alert, enterococcus durans, enterococcus faecalis, enterococcus faecium, enterococcus gallinarum, linezolid (lzd), multi-drug resistance (mdr), vancomycin resistant enterococcus (vre), vitek 2 system

## Abstract

Background

*Enterococcus*, once benign intestinal flora, has transformed into formidable nosocomial pathogens as a result of the accelerated emergence of antibiotic resistance represents a major global health challenge, particularly within hospital settings. *Enterococcus* has grown more prevalent in nosocomial infections, such as urinary tract infections (UTIs), surgical site infections (SSIs) and bacteremia. The potential emergence of vancomycin-resistant *Enterococcus *(VRE) strains further complicates treatment choices for multi-drug resistant (MDR) infections. This study evaluated the magnitude of *Enterococcus* infections and their antibiotic resistance patterns in a tertiary care hospital.

Material and methods

A laboratory-based cross-sectional study was conducted from January 2023 to December 2023 (one year) at Krishna Hospital & Medical Research Centre, Karad, India. A total of 189 enterococcalisolates were identified from various clinical specimens, including urine, blood, pus, and other samples. These isolates were subjected to identification and antimicrobial susceptibility testing using the automated VITEK 2 (bioMérieux SA, Marcy-l’Étoile, France) system. VanA and VanB phenotypes were detected based on minimum inhibitory concentration (MIC) values using the VITEK 2 Advanced Expert System (AES) system (bioMérieux SA). Statistical analysis was done using Statistical Package for the Social Sciences (IBM SPSS Statistics for Windows, IBM Corp., Version 21.0, Armonk, NY).

Results

Among 189 culture-positive enterococcal isolates, the majority were obtained from urine 144 (76%), followed by blood 17 (9%), pus 12 (6%), etc. A larger proportion of these isolates were from female patients 97 (51%) aged over 60 years. A substantial proportion of these isolates originated from in-patient departments (IPD) 178 (94.2%) with intensive care units contributing the highest number 114 (60%) and out-patient departments (OPD) 11 (5.8%). The highest prevalence of infection was observed among patients with a hospital stay of 8 to 14 days (32.3%). *E. faecium *(57.7%) and *E. faecalis *(39.6%) were the predominant species with *E. faecium *displaying significant resistance to benzylpenicillin (96%) and nitrofurantoin (94%) whereas *E. faecalis *showed higher resistance to high-level gentamicin (80%). *E. gallinarum *(1.6%)showed complete resistance to benzylpenicillin (100%) and moderate resistance to nitrofurantoin (67%), and high-level gentamicin (67%). Conversely, *E. durans* showed complete resistance (100%) to both nitrofurantoin and benzylpenicillin. Among 31 VRE isolates, 16 (52%) showed the VanA and 15 (48%) showed the VanB phenotype.

Conclusion

Antimicrobial resistance among *E. faecium *and *E. faecalis*, particularly to benzylpenicillin, high-level gentamicin and nitrofurantoin along with the emergence of resistant species like *E. durans *and *E. gallinarum *underscores the urgent need for vigilant antimicrobial stewardship and continuous surveillance of the growing threat of MDR enterococci in clinical settings.

## Introduction

The accelerated emergence of antibiotic resistance among prevalent pathogens represents a formidable global health challenge, particularly within hospital settings. *Enterococcus* is one of the organisms that has grown prevalent in nosocomial infections, such as urinary tract infections (UTIs), surgical site infections (SSIs), and bacteremia [1,2]. The fact that, unlike *Streptococcus*, genus *Enterococcus* is intrinsically resistant to many antimicrobials such as penicillins, ampicillin, cephalosporins, semi-synthetic penicillins, aminoglycosides (with low-level resistance), clindamycin, and trimethoprim-sulfamethoxazole pose significant therapeutic hurdles [3]. The potential of vancomycin-resistant *Enterococcus* (VRE) strains to transmit resistance genes to *Staphylococcus aureus *is a specific cause for concern as it further complicates treatment choices for multi-drug resistant (MDR) infections [4,5].

Cell wall inhibitors and aminoglycosides have been used in conjunction to treat *Enterococcus *infections. Nevertheless, conventional synergistic antibiotic regimens are no longer effective due to the rise of high-level aminoglycoside resistance (HLAR) and decreased susceptibility to β-lactam antibiotics and vancomycin [6,7]. Since Uttley et al. in the year 1988 initially reported the rising frequency of VRE, treating these infections has become increasingly challenging. The effectiveness of traditional treatment plans is called into question by this resistance profile, which raises serious questions regarding the future of antimicrobial therapy in hospital settings [8].

Both *E. faecalis* and *E. faecium* are the most prevalent species linked to infections in humans. *E. faecium* is more resistant and isolated more frequently, especially in hospital settings [9,10]. These organisms are resilient to extreme environments, including high salt concentrations and fluctuating pH levels, as well as their propensity to adhere to hospital surfaces, all contribute to their status as tenacious hospital-acquired pathogens, which exacerbates the problem [11-14].

Given the increase of enterococcal infections in tertiary care hospitals, there is a pressing need for ongoing surveillance of antimicrobial resistance patterns in *Enterococcus *species, particularly in regions where data may be limited [15,16]. This study was undertaken in a tertiary care hospital in the Western region of Maharashtra, India to determine the magnitude of *Enterococcus *infections and their antimicrobial resistance patterns. The findings will offer valuable insights into local enterococcal infections and guide targeted therapeutic interventions to address this critical clinical challenge.

## Materials and methods

A laboratory-based cross-sectional study was performed at the Department of Microbiology, Krishna Hospital & Medical Research Centre, Karad, India over a period of one year from January 2023 to December 2023. The ethical clearance for this study was granted by the Institutional Ethics Committee, Krishna Institute of Medical Sciences (KIMS) issued approval KVV/IEC/05/2024 under protocol number 338/2023-2024. This study included 189 enterococcal isolates. A pre-structured proforma was utilized to document the patient’s demographic data and clinical information including age, sex, hospital stay duration, and location. All clinical samples collected during the study period included urine, blood, pus, cerebrospinal fluid (CSF), vaginal swab, body fluids, sputum, tissues, etc. from the population of patients of all age groups and both sexes with enterococcal infection. The samples were transported immediately to the microbiology laboratory and processed immediately after collection.

Isolation of bacterial colony

For blood cultures, 5 to 10 mL of blood for adults and 2 to 3 mL for pediatric patients were inoculated into blood culture bottles (BACT/ALERT FA Plus for adults and BACT/ALERT PF Plus for pediatrics) and monitored for up to five days using the BACT/ALERT 3D-60 (bioMérieux SA, Marcy-l’Étoile, France) automated blood culture system. One drop from each positive bottle was plated on a standard bacteriological culture media such as MacConkey agar, Chocolate agar, and Blood agar. On the other hand, urine, pus, sputum, and others were directly inoculated on standard bacteriological media. All culture plates were incubated at 37°C for 18 to 24 hours.

Identification and antimicrobial susceptibility testing

The bacteriological identification and antimicrobial susceptibility test were performed with VITEK 2 COMPACT (bioMérieux SA) using a gram-positive identification (ID-GP) card and susceptibility card (AST-628) according to the standard instructions described below.

Every bacterial suspension was prepared from pure cultures of bacteria cultured on standard bacteriological media. Bacterial cells were suspended in 3 mL of 0.45% sterile sodium chloride solution. The turbidity of the suspension was adjusted to 0.5 McFarland standard using Densicheck (bioMérieux SA). The results of antimicrobial susceptibility were interpreted according to Clinical and Laboratory Standards Institute (CLSI) M100 34^th^ edition. *E. faecalis* ATCC 29212 was used as a quality control strain.

Following antibiotics were included in the study: benzylpenicillin (P), nitrofurantoin (FT), ciprofloxacin (CIP), levofloxacin (LEV), high-level gentamicin (HLG), linezolid (LNZ), tigecycline (TGC), tetracycline (TE), teicoplanin (TEC) and vancomycin (VA).

VanA and VanB are the most prevalent vancomycin-resistant phenotypes in *E. faecalis* and *E. faecium with* VanA phenotype being the most common and clinically relevant form of vancomycin resistance. Phenotypic detection of VanA and VanB was performed based on minimum inhibitory concentration (MIC) values using the Advanced Expert System (AES) system, a crucial component of the VITEK 2 system that provides fingerprint-based recognition of bacterial phenotypes and resistance mechanisms. The VanA phenotype has strong resistance to vancomycin and teicoplanin, with vancomycin MIC ≥ 64 µg/mL and teicoplanin MIC ≥ 16 µg/mL, respectively. In contrast, the VanB phenotype has varied resistance to vancomycin but is susceptible to teicoplanin. Vancomycin's MIC in VanB strains ranges from 4 µg/mL to ≥ 1000 µg/mL, while teicoplanin's MIC is ≤ 8 µg/mL [1,4].

## Results

In the laboratory of the Department of Microbiology, Krishna Hospital & Medical Research Centre, Karad, 189 culture-positive *Enterococcus*, isolated from various clinical samples, were studied. Out of these 189 isolates, 109 (57.7%) were identified as *E. faecium*, 75 (39.6%) as *E. faecalis*, three (1.6%) as *E. gallinarum* and two (1.1%) as *E. durans* (Table 1).

**Table 1:** Comparative Distribution of Enterococcus Species in Isolated Strains

*Enterococcus* species	Number (n)	Percentage (%)
E. faecium	109	57.7
E. faecalis	75	39.6
E. gallinarum	3	1.6
E. durans	2	1.1
Total	189	100

**Figure 1:**
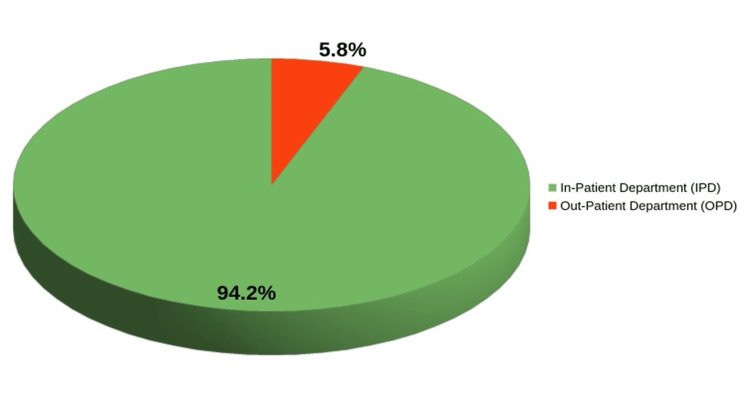
Source Distribution of Isolates by Clinical Setting

Significantly, the majority of these isolates originated from the in-patient department (IPD) (178, 94.2%) with the intensive care unit (ICU) contributing the highest number (114, 60%), and out-patient department (OPD) (11, 5.8%) (Figure 1).

**Figure 2:**
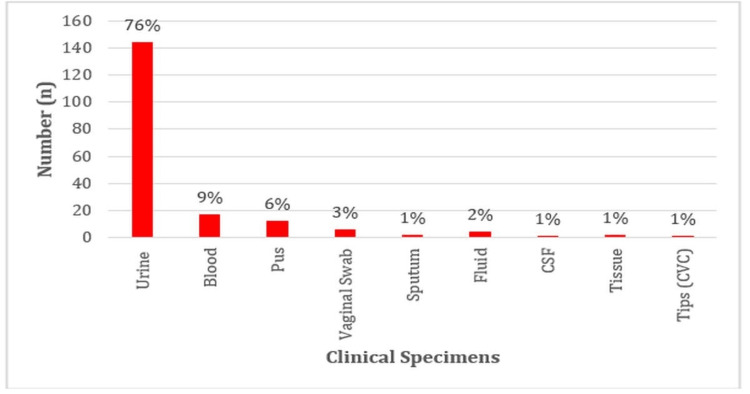
Distribution of Enterococcal Isolates in Various Clinical Specimens CSF: cerebrospinal fluid; CVC: central venous catheter

Urine samples were the most common source of these isolates (144, 76%), followed by blood (17, 9%), pus (12, 6%), and a variety of other sources, including tissue samples, vaginal swabs, sputum, bodily fluids, CSF, and catheter tips (Figure 2).

**Figure 3:**
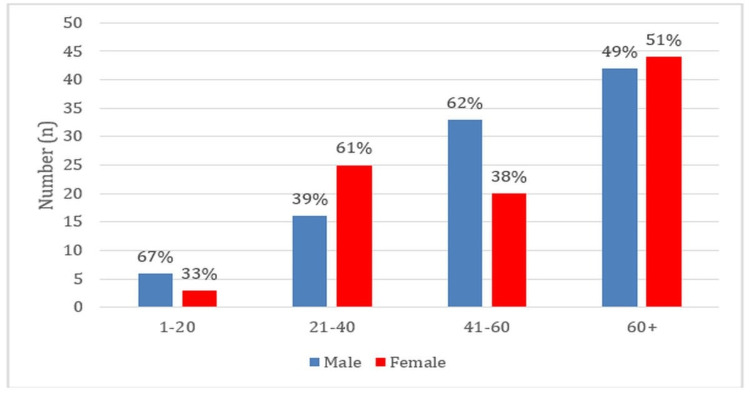
Distribution of Patients by Age and Gender With Isolated Enterococcus

Patients who spent eight to 14 days in the hospital had the greatest infection rate of 32.3%. The majority of isolates were predominantly procured from individuals over 60 years old, with female patients (97, 51%) outnumbering male patients (92, 49%) (Figure 3).

**Figure 4:**
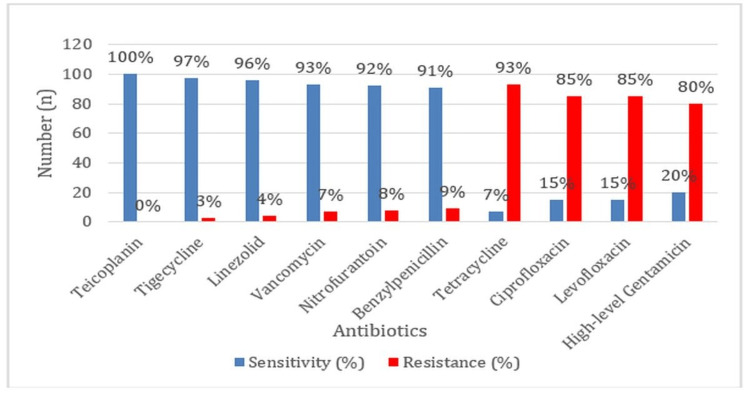
Antibiotic Sensitivity and Resistance Profiles of Enterococcus faecalis

*E. faecalis* was highly sensitive to teicoplanin (100%) followed by tigecycline (97%), linezolid (96%), vancomycin (93%), nitrofurantoin (92%), and benzylpenicillin (91%), while significant resistance was seen to tetracycline (93%), ciprofloxacin (85%), levofloxacin (85%), and high-level gentamicin (80%) (Figure 4).

**Figure 5:**
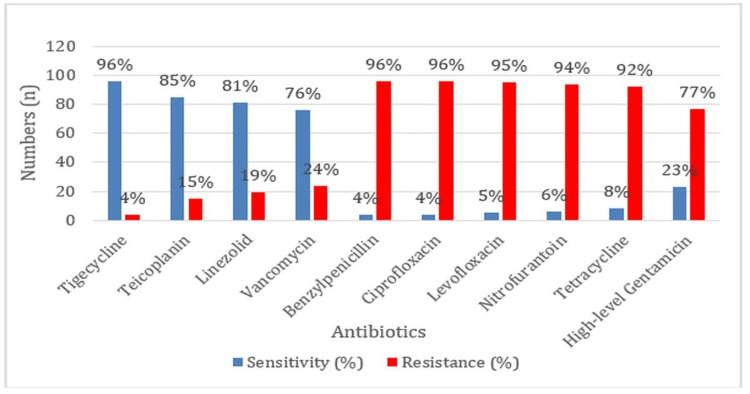
Antibiotic Sensitivity and Resistance Profiles of Enterococcus faecium

In contrast to its high sensitivity to tigecycline (96%), teicoplanin (85%), linezolid (81%), and vancomycin (76%), *E. faecium* demonstrated a significant degree of resistance to benzylpenicillin (96%), ciprofloxacin (96%), levofloxacin (95%), nitrofurantoin (94%), tetracycline (92%), and high-level gentamicin (77%) (Figure 5).

**Figure 6:**
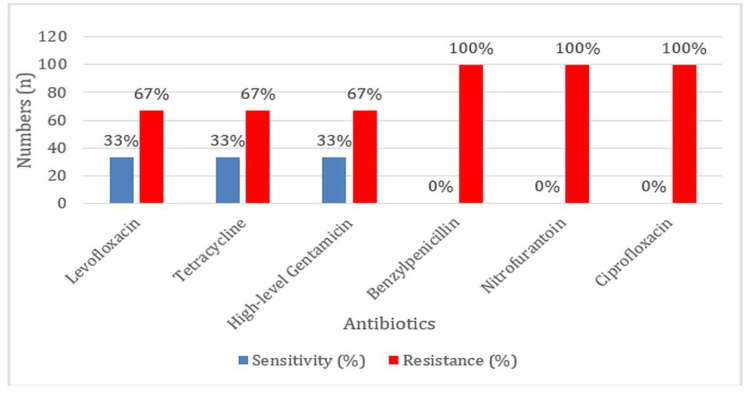
Antibiotic Sensitivity and Resistance Profiles of Enterococcus gallinarum

A worrying degree of resistance was shown by *E. gallinarum* with 100% resistance to benzylpenicillin, nitrofurantoin, and ciprofloxacin. High-level gentamicin, levofloxacin, and tetracycline exhibited low sensitivity, with only 33% of isolates responding (Figure 6).

**Figure 7:**
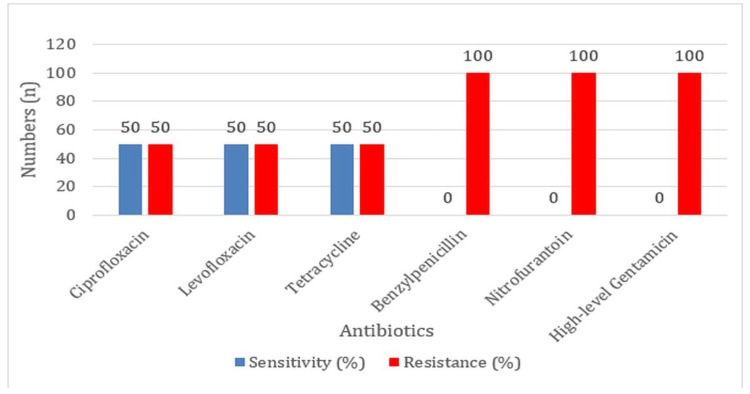
Antibiotic Sensitivity and Resistance Profiles of Enterococcus durans

Moreover, resistance levels to nitrofurantoin, high-level gentamicin, and benzylpenicillin were very high, with 100% resistance observed for *E. durans* and 50% sensitivity to ciprofloxacin, levofloxacin, and tetracycline (Figure 7).

**Figure 8:**
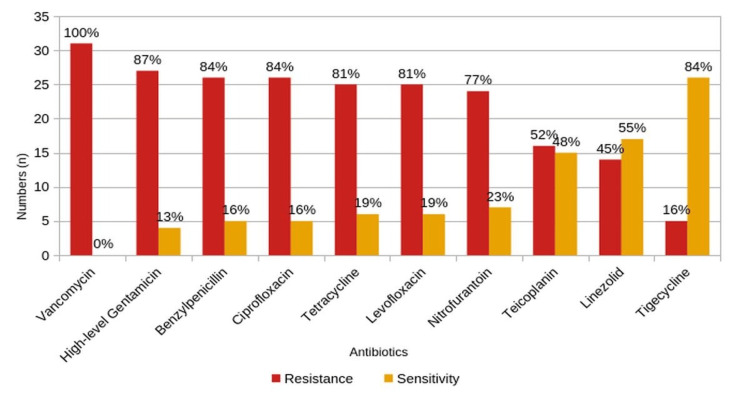
Antimicrobial Resistance and Susceptibility Patterns of Vancomycin-Resistant Enterococcus (VRE) Isolates

Among the 189 *Enterococcus* isolates, 31 (16%) were VRE showing the most sensitivity to tigecycline (84%) and linezolid (55%). The highest rates of resistance were found for vancomycin (100%), high-level gentamicin (87%), benzylpenicillin (84%), ciprofloxacin (84%), levofloxacin (81%), tetracycline (81%), and nitrofurantoin (77%) (Figure 8).

The 31 VRE isolates included five *E. faecalis* and 26 *E. faecium*, of which 16 (52%) had the VanA phenotype and 15 (48%) had the VanB phenotype (Table 2).

**Table 2:** Phenotypic Distribution (VanA and VanB) Among Enterococcus Species Based on Vancomycin and Teicoplanin MIC Profile n: number; µg/mL: microgram per millilitre; ​​*E. faecium*: *Enterococcus faecium; E. faecalis*: *Enterococcus faecalis; *MIC: minimum inhibitory concentration

Phenotypes	*Enterococcus *species	Number of isolates	Vancomycin MIC ≥ 32 (µg/mL)	Teicoplanin MIC (µg/mL)	
				≤8	≥32
VanA	E. faecium	16	16	0	16
VanB	E. faecium	10	10	10	0
VanB	E. faecalis	5	5	5	0

## Discussion

The rise of antimicrobial resistance in bacteria to modern medicine remains one of the biggest problems today. A striking capacity of Gram-positive *Enterococcus*, to express, acquire and magnify genetic material that facilitates antibiotic resistance is apparent. Investigation of a range of nosocomial diseases has shown high-level resistance patterns, especially in tertiary care hospitals in India. With this trend, it highlights the need for strict infection control methods to stop these infections from spreading [1-4].

In the present study, the most prevailing species in the 189 isolates was *E. faecium*, at a prevalence of 57.7%, followed by 39.6% with *E. faecalis*, 1.6% with *E. gallinarum*, and 1.1% with *E. durans*. This finding corresponds with the study by Mahajan M et al. in 2024, which also reported a high prevalence of *E. faecium* (53.33%) in Indian tertiary care settings [16].

Enterococcal infection in the patients of the IPD was relatively high (178, 94.2%), in which 114 (60%) isolates were from the ICU and 11 (5.8%) were from the OPD, indicating that the IPD has a high infection rate within which ICU patients are particularly susceptible to infections related to invasive interventions and increased exposure to broad-spectrum antibiotics. This observation aligns with extensive research by Mahajan M et al. in 2024 which reported 96.66% of isolates originated from IPD [16]. Thakan K and Vij A in 2022 also reported that a significant proportion of enterococcal infections were found in the IPD (69%) and 31% in the OPD [7].

Urine samples constituted 76% of the isolates, followed by blood (9%) and pus (6%). This present study is in agreement with the study by Backiam A et al. in 2023 which cited that UTIs and the bloodstream are the commonest clinical manifestations of *Enterococcus* infection [8].

A higher rate (32.3%) of infection was observed in patients more than 60 years of age, with infection rates closely associated with longer hospital stay days of eight to 14 days, strongly indicating the increased risks associated with longer hospital stays. This trend is concordant with the study by Rana D and Sande S in 2020 with an infection rate of 37% [5].

Furthermore, the majority of these isolates were from females (51%) compared to males (49%). This female gender preponderance in the frequency of infection suggests potential sex-related differences that warrant further study, aligning with the findings by Backiam A et al. in 2023, which reported a female prevalence of 60% and a male prevalence of 40% [8]. Such insights are crucial for the clinical management and targeted intervention to reduce the incidents of enterococcal infections in healthcare settings.

The susceptibility and resistance profiles reported in this study for *E. faecalis* are comparable to earlier reports from Rana D and Sande S in 2020 and Said HS and Abdelmegeed ES in 2019 [5,14]. The results from the present study indicate significant susceptibility to teicoplanin (100%), linezolid (96%), and vancomycin (93%), comparable to the respective values reported by Rana D and Sande S, as 91.5%, 97.2%, and 88.6% respectively [5]. Resistance to tetracycline (93%), ciprofloxacin (85%), levofloxacin (85%), and high-level gentamicin (80%) complies with the study by Said HS and Abdelmegeed ES in 2019, who reported similar rates of resistance in tetracycline (93%), ciprofloxacin (84.5%), levofloxacin (88.7%), and high-level gentamicin (80.3%) [14]. These studies show that the susceptibility and resistance patterns are comparable both at the regional and study-specific levels.

In this study, *E. faecium* showed a higher rate of resistance to benzylpenicillin (96%), ciprofloxacin (96%), levofloxacin (95%), nitrofurantoin (94%), and high-level gentamicin (77%) and showed high susceptibility to tigecycline (96%), teicoplanin (85%), linezolid (81%), and vancomycin (76%). These results were in agreement with other studies, such as Yadav KR and Jha BK in 2021, with similar susceptibility to linezolid (81.3%) and resistance to levofloxacin (90.6%) and high-level gentamicin (78.1%) [15]. Das AK et al. in 2022 and Karna A et al. in 2019 also showed the same susceptibility to tigecycline at 90%, teicoplanin at 80%, and vancomycin at 70% [2,3]. However, nitrofurantoin resistance in this study is 94%, which is higher than that of the report (45%) by Das AK et al. in 2022 [2]. This could be explained by regional differences in prescribing and infection control measures. Targeted antimicrobial stewardship programs may benefit this pattern of resistance by curtailing the overuse of nitrofurantoin and improving infection control to limit the spread of resistant strains within healthcare settings.

The resistance profiles of *E. gallinarum* in the current study showed high resistance to benzylpenicillin (100%). The results obtained by this study agreed with those reported in the earlier study reported by Yadav KR and Jha BK in 2021, where it was highlighted, that MDR is not limited to such more common species [15]. *E. durans* also exhibits an acceptably high level of resistance to nitrofurantoin (100%), an antibiotic that is commonly used in the treatment of UTIs, high-level gentamicin (100%), and benzylpenicillin (100%). This makes surveillance on resistance patterns in these two species even more vital as their increasingly higher resistance level would pose serious challenges to treating the infections, especially UTIs, for which nitrofurantoin is a primary therapeutic agent.

The automated VITEK 2 Compact with AES identified 16% of the 189 *Enterococcus* isolates as VRE showing 84% sensitivity to tigecycline and 55% to linezolid. These results align with Das AK et al. in 2022 which reported a higher sensitivity for tigecycline (70%) and linezolid (95%) against VRE. This high efficacy is likely due to tigecycline’s binding to the 30S ribosomal subunit and linezolid’s targeting of 23S rRNA in the 50S subunit, thereby inhibiting the process of protein synthesis with very limited pathways for developing resistances as noted [2].

The present study identified 31 VRE isolates with both VanA (51.7%) and VanB (48.3%) phenotypes. Scenarios like these are difficult to manage in terms of standard first-line antibiotics. Earlier studies by Yadav RK and Agarwal L in 2022 reported a prevalence of VanA at 71.4% and VanB at 28.5%, suggesting a greater complexity of resistance in general [4]. The presence of both the VanA and the VanB phenotypes with complex resistance profiles prompts the development of effective antimicrobial stewardship with highly monitored management strategies and remedial therapies to combat this ever-growing trend of VRE.

## Conclusions

This study highlights an increasingly complicating problem of MDR *Enterococcus* species found in Western Maharashtra tertiary care hospitals. Its predominance goes to the *E.* *faecium*, which demonstrates considerable resistance against beta-lactams, fluoroquinolones, aminoglycosides, and glycopeptides groups of antibiotics. The presence of VRE strains with the VanA and VanB phenotypes complicates the treatment further, especially in more severe illnesses or ICU patients, who have a higher risk of developing nosocomial infections. Tigecycline and linezolid have been shown to be sensitive and effective against VRE because of their action mechanisms that target bacterial protein synthesis uniquely. These results highlight the need for locally adapted treatment approaches, better infection control measures, and intense antimicrobial stewardship. Furthermore, the increased prevalence and notable resistance trends among *E. faecium*, *E. faecalis*, *E. gallinarum*, and *E. durans* underscores the need to continue surveillance and research into alternative therapeutic approaches to combat this critical threat of enterococci that are increasingly becoming resistant in healthcare settings.
